# Genome-Wide Screen for *Mycobacterium tuberculosis* Genes That Regulate Host Immunity

**DOI:** 10.1371/journal.pone.0015120

**Published:** 2010-12-10

**Authors:** Aimee M. Beaulieu, Poonam Rath, Marianne Imhof, Mark E. Siddall, Julia Roberts, Dirk Schnappinger, Carl F. Nathan

**Affiliations:** 1 Department of Microbiology and Immunology, Weill Cornell Medical College, New York, New York, United States of America; 2 Division of Invertebrate Zoology, American Museum of Natural History, New York, New York, United States of America; University of Hyderabad, India

## Abstract

In spite of its highly immunogenic properties, *Mycobacterium tuberculosis* (Mtb) establishes persistent infection in otherwise healthy individuals, making it one of the most widespread and deadly human pathogens. Mtb's prolonged survival may reflect production of microbial factors that prevent even more vigorous immunity (quantitative effect) or that divert the immune response to a non-sterilizing mode (qualitative effect). Disruption of Mtb genes has produced a list of several dozen candidate immunomodulatory factors. Here we used robotic fluorescence microscopy to screen 10,100 loss-of-function transposon mutants of Mtb for their impact on the expression of promoter-reporter constructs for 12 host immune response genes in a mouse macrophage cell line. The screen identified 364 candidate immunoregulatory genes. To illustrate the utility of the candidate list, we confirmed the impact of 35 Mtb mutant strains on expression of endogenous immune response genes in primary macrophages. Detailed analysis focused on a strain of Mtb in which a transposon disrupts Rv0431, a gene encoding a conserved protein of unknown function. This mutant elicited much more macrophage TNFα, IL-12p40 and IL-6 in vitro than wild type Mtb, and was attenuated in the mouse. The mutant list provides a platform for exploring the immunobiology of tuberculosis, for example, by combining immunoregulatory mutations in a candidate vaccine strain.

## Introduction

Mtb is one of the most potent admixtures of antigens and adjuvants known. In the laboratory, heat-killed Mtb emulsified in oil and water (Freund's complete adjuvant) helps elicit high-titer antibodies in animal models. Infection by Mtb elicits robust and long-lasting T and B cell responses to numerous Mtb antigens. Human T-cell responses to Mtb are so vigorous that injection of the dead bacillus into an already infected individual leads to inflammation-mediated tissue necrosis, a response called the Koch phenomenon [1].

Yet human immunity frequently fails to eradicate Mtb infection and an estimated one-third of the global population is latently infected for life. Of these, 5–10% with no known impairment of immunity will go on to develop active tuberculosis and infect an average of 10 to 15 others [2]. When cured, such individuals remain susceptible to re-infection and can again develop active tuberculosis [3], [4], [5]. Mtb appears to have evolved to elicit an immune response too weak to fully clear infection but paradoxically strong enough, in hosts with active disease, to liquefy lung tissue and cause an infectious cough that allows transmission to new individuals. These circumstances pose a major question for immunobiology with significant implications for vaccinology: How does Mtb sculpt the host immune response to be quantitatively large but often qualitatively inadequate?

Answering this question requires a better understanding of how Mtb modulates immunity. Murine models of infection recapitulate a number of key features of the human disease and allow the use of genetically modified bacteria and hosts to identify determinants of pathogenicity and susceptibility. In one such model, genetically resistant mice inhale small numbers of bacilli that are phagocytosed by macrophages in the pulmonary alveoli. The bacilli are transported into the interstitium and taken up by resident or recruited monocytes, macrophages, dendritic cells and neutrophils [6]. Dendritic cells carry Mtb to draining lymph nodes, where naïve T cells are activated. Before, during or despite T cell activation, the bacteria replicate and escape into the efferent lymph, which carries them to the bloodstream for dissemination throughout the body, where most are taken up by macrophages. In people, disease can emerge at any time in any site, but most often appears years later in the lung.

Following priming and clonal expansion in lymph nodes, mouse Mtb-specific T cells traffic to the lung at ∼2–3 weeks post-infection [7], [8], [9]. The onset of a Th1-dominated immune response is marked by elevation of transcripts for IL-2, IL-12p40, IFNγ, TNFα and iNOS by 2 to 3 orders of magnitude [10], [11]. Cytokines, in particular IFNγ, synergize with products of Mtb to activate infected macrophages [12]. These events coincide with and are required for the abrupt cessation in bacterial outgrowth at ∼3 weeks post-infection, whereupon replication and death rates reach an equilibrium and bacterial numbers are maintained at near steady-state levels [13]. Nonetheless, inflammation escalates over many months until the mouse succumbs to respiratory insufficiency [14].

Presumably, early innate responses shape the adaptive immune response to Mtb. As a primary reservoir for Mtb in vivo, macrophages are crucial for controlling bacterial growth but also influence the function of other cells in the immune system, including T- and B-lymphocytes [15], [16]. Recent “forward genetics” studies, aimed at identifying novel innate immunity pathways and factors that modulate the host-pathogen interaction, have unearthed essential roles for macrophage autophagy and production of immunomodulatory lipids in controlling Mtb disease [17], [18], [19]. We focused here on the early encounter of macrophages with Mtb and included IFNγ to model the influence of NK cells and γδ T cells that produce IFNγ during the earliest days of infection [20], [21], as do early-arising αβ T cells. The literature describes the impact of several dozen Mtb genes or gene products on early responses of macrophages [22], [23]. To expand that knowledge we systematically assessed macrophage responses to a large panel of loss-of-function mutants of Mtb.

## Results

### Development of Immunoreporter Macrophage Clones

To identify mutations in Mtb that lead to dysregulation of the macrophage immune response, we screened a library of 10,100 Mtb mutants generated by transposon insertion mutagenesis. The same library has provided valuable hits in previous screens [24], [25]. With its dependence on expansion of mutant clones, the library lacks inactivating mutations in genes essential for in vitro growth, but otherwise it approaches the ideal of representing loss of function mutations in most Mtb genes. Ideally we would globally evaluate macrophage responses to each Mtb mutant. However, safety constraints prevented us from generating large volumes of infectious waste as would be required by multiplexed ELISAs, and cost considerations precluded profiling the macrophage transcriptome over 10,000 times. We therefore limited the survey of the macrophage response to 12 host genes whose expression could be detected microscopically using fluorescent proteins as reporters.

**Figure 1:**
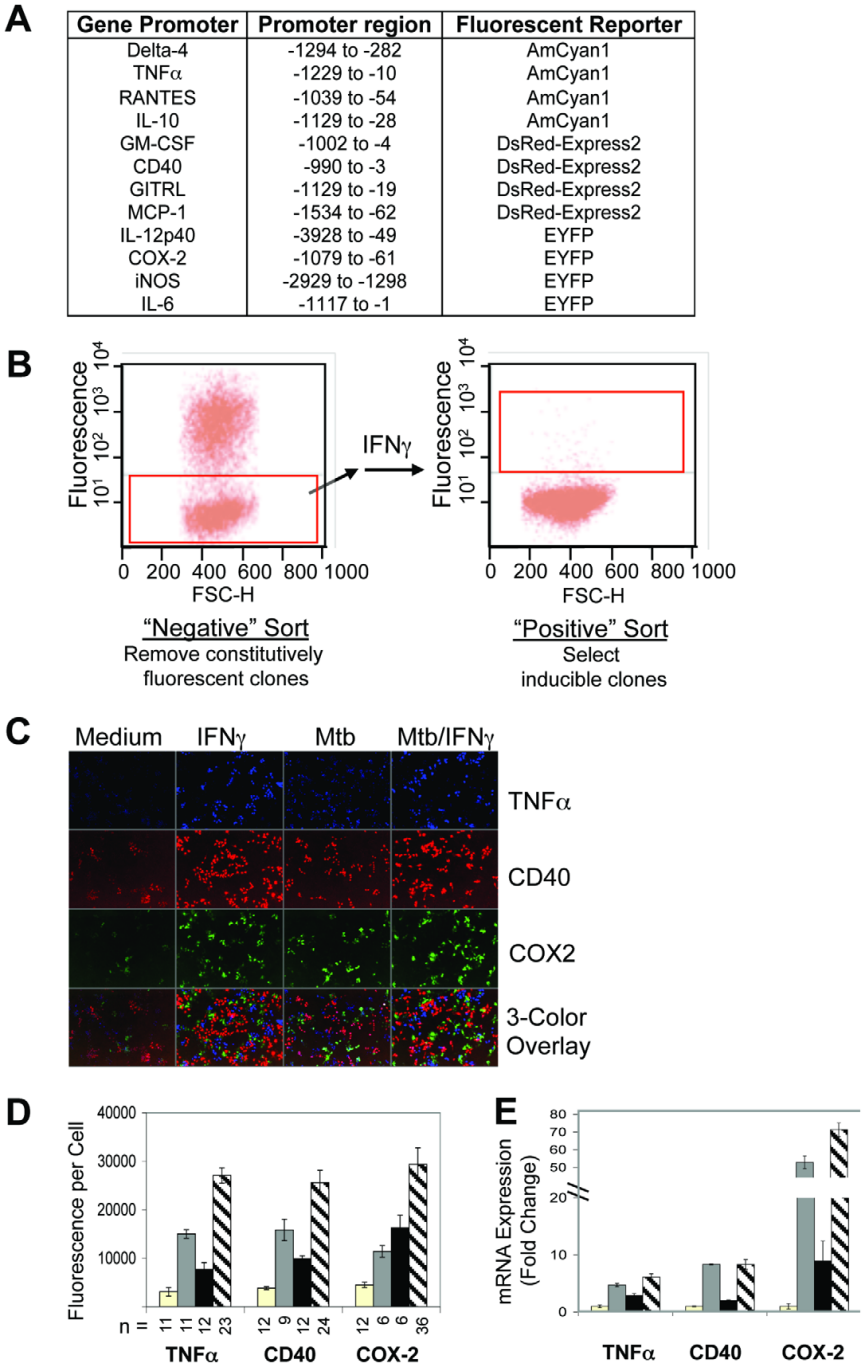
Generation and Validation of Immunoreporter Macrophage Clones. (**A**) RAW 264.7 cells were transfected with constructs in which the promoter of a host gene was used to drive expression of a fluorescent reporter protein. The promoter region and 5′-UTR are specified relative to the “ATG” start codon of each gene. (**B**) Stably transfected IR populations were sorted by FACS to isolate cells with low baseline fluorescence (red box on left panel), expanded in vitro, then stimulated with IFNγ. Cells that upregulated fluorescence (red box on right panel) were sorted again to yield clonal, inducible IR macrophage clones. (**C**) IR macrophages expressing the promoter-reporter TNFα_AmCyan, CD40_DsRed, or COX2_YFP constructs were seeded together, then treated with WT Mtb (MOI ∼10), IFNγ, or both for 24 hrs. Fluorescence induction was assessed using automated fluorescence microscopy in the three distinct wavelengths. The bottom row represents a digital overlay of the three top rows. (**D**) Quantitation of (C) using Metamorph software. Data represent means ± SD of numbers (n) of independent wells indicated. (**E**) WT RAW 264.7 cells were treated as in C. At 8 hrs, gene induction was evaluated by qRT-PCR. Expression levels are relative to *gapdh* and are expressed as fold change relative to treatment with medium. Data represent means ± SD of triplicate wells.

We selected the host genes (Figure 1A) by the following criteria. Pilot studies, in conjunction with published reports, confirmed that expression of all 12 genes is transcriptionally regulated in mouse macrophages in response to Mtb infection. Most are differentially regulated during tuberculosis in human subjects and, with the exception of Delta-4 and GITRL, targeted disruption of these genes has been reported to impart a phenotype in mice with experimental tuberculosis. Finally, they represented diverse molecular classes – cytokines (TNF, IL-12p40, IL-6, IL-10, GM-CSF), chemokines (RANTES, MCP-1), enzymes (iNOS, cyclooxygenase [COX] 2) and cell-surface co-stimulatory proteins (Delta-4, CD40, GITRL) – with known roles in immune responses to infection.

We stably transfected murine macrophage-like RAW 264.7 cells with promoter-reporter constructs in which a promoter region of each host immune-response gene drives expression of one of three fluorescent proteins (AmCyan, DsRed or YFP) (Figure 1A). Each of the twelve Immunoreporter (IR) macrophage lines was subjected to clonal selection as follows. Unstimulated cells were sorted by flow cytometry to isolate populations with minimal resting fluorescence. From this group, cells that manifested high levels of fluorescence when stimulated with IFNγ (Figure 1B) were expanded as individual clones. Finally, by microscopy (Figure S1) we identified clones from each line that expressed homogeneously low fluorescence at baseline and homogeneously high fluorescence upon stimulation with IFNγ or with LPS.

Stimulation of the IR macrophage cell clones with IFNγ, Mtb, or Mtb + IFNγ confirmed that fluorescence induction (Figure 1C, D) qualitatively reflected mRNA expression patterns for the native gene products (Figure 1E). Further, fluorescence levels increased along with increasing multiplicity of infection (MOI) (data not shown). As a positive control for the ability of these IR clones to resolve differences in stimulation induced by an Mtb mutant compared to wild type (WT) Mtb, we infected the macrophages with a mutant in *mce1* that was reported to induce sub-normal levels of IL-6 and MCP-1 [26]. Indeed, the mutant elicited lower levels of fluorescence in the IL-6_YFP and MCP-1_DsRed IR clones than WT Mtb, mirroring reduced secretion of IL-6 and MCP-1 protein in wild-type RAW 264.7 cells (Figure S2A & S2B).

### Primary Screen

**Figure 2:**
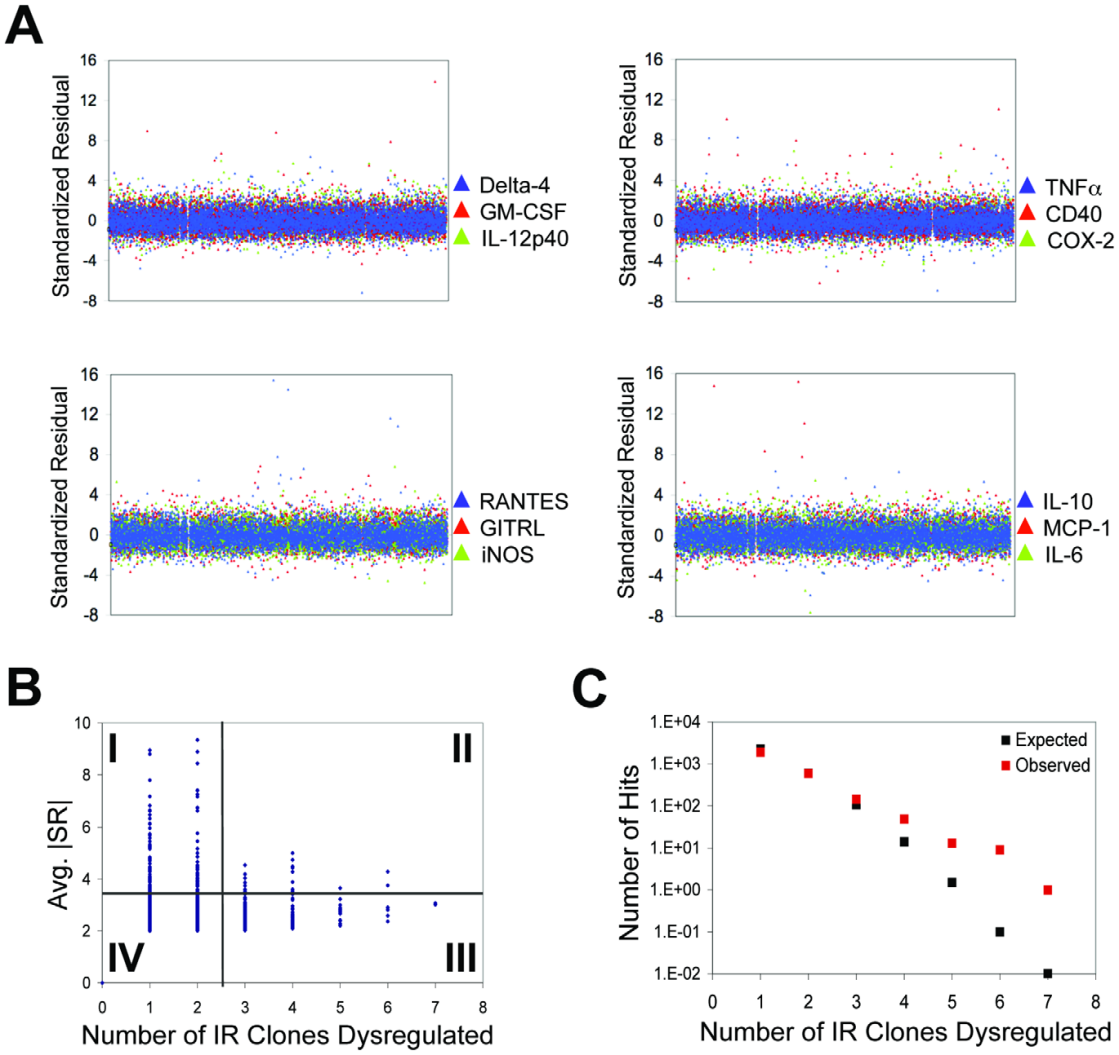
Results of the Primary Screen. (**A**) The SR of the response induced by each mutant, represented by points across the x-axis. Data points are color-coded according to the IR line in which the response was measured. (**B**) Hits (y-axis), defined as mutants that induced responses with an avg. |SR| >2, were stratified according to the number of IR clones they dysregulated (x-axis). Mutants were categorized into four groups: (I) those that strongly dysregulated fluorescence (avg. |SR| >3.5) in only 1 or 2 IR clones; (II) those that strongly dysregulated fluorescence in more than 2 IR clones; (III) those that moderately dysregulated fluorescence (avg. |SR|  = 2–3.5) in more than 2 IR clones; and (IV) those that moderately dysregulated fluorescence in 1 or 2 IR clones. (**C**) The number of hits (|SR| >2) is depicted as a function of the number of IR clones dysregulated by each mutant. The numbers expected by chance (black) are compared to the numbers observed in the primary screen (red). Divergence between the observed and expected values was greatest when ≥4 IR clones were dysregulated.

Next we plated the 12 IR macrophage clones in 4 sets of 3. Thus, each well of an assay plate contained one IR clone signaling the expression of a given promoter via AmCyan, another announcing the activity of a different promoter via DsRed and a third reporting on yet another promoter via YFP. We infected each such well in each set with one of the 10,100 Mtb mutants and monitored alterations in fluorescence induction by automated microscopy. The use of pooled IR macrophage clones cut down the time and cost of screening by a factor of 3. Based on pilot experiments that indicated a better signal-to-noise ratio following IFNγ-costimulation, we reduced the burden of screening by another factor of 2 by omitting a no-IFNγ set and using IFNγ-costimulation throughout. Because the insertion of a transposon can affect Mtb biology, we reasoned that the most relevant baseline was the average response induced by all 10,1000 mutants. We considered a mutant to be a candidate hit if it elicited fluorescence levels differing from this average response to a statistically robust extent (see below). Each fluorescence level was characterized by its standardized residual (SR), a measure of the number of standard deviations separating it from the mean (Figure 2A).

Applying a cut-off of SR >2.0 or <−2.0 yielded 2712 unique transposon mutants that induced altered fluorescence in one or more of the 12 IR clones (Figure 2A–B). The majority (2492; 91.9%) of these Mtb mutants dysregulated responses in only one or two of the IR clones (1885 and 607 Mtb mutants, respectively). However, these numbers were not greater than expected by chance, encouraging us to apply more robust statistical criteria (Figure 2C). Application of a cut-off value of |SR| >3.5 reduced the number of mutants that qualified as hits in one or two IR clones to 99 (Table S1) and 46 (Table S2), respectively (quadrant I of Figure 2B). Using the cutoff of |SR| >2.0, 210 mutants elicited aberrant responses in three or more IR clones, while only 125 such hits would be expected by chance. Differences between the observed number of hits and the number expected by chance were most dramatic for mutants that dysregulated four or more IR clones (Figure 2C). The 210 mutants exceeding this level for at least three IR clones could be further subdivided into 22 that elicited highly aberrant responses in three or more IR lines (average |SR| >3.5; quadrant II in Figure 2B and Table S3) and 188 that elicited moderately aberrant responses in three or more IR lines (average 2.0< |SR| <3.5; quadrant III in Figure 2B and Table S4).

### Secondary Screen

**Figure 3:**
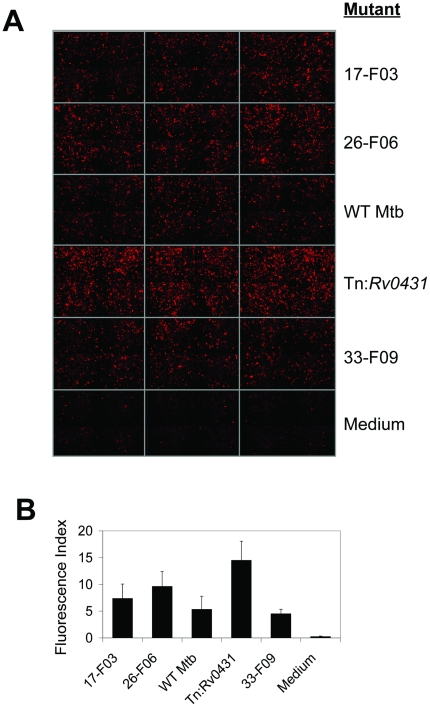
Illustration of Hit Confirmation in the Secondary Screen. (**A**) GITRL_DsRed IR macrophages were infected with WT Mtb or mutants identified in the primary screen (MOI ∼10). Fluorescence induction was assessed at 24 hrs by automated fluorescence microscopy. Images from three replicate wells per condition are shown. (**B**) Quantitation of (A) using Metamorph software. Data represent means plus SD of 5–6 independent wells per condition.

The Mtb mutants chosen for further study (see below) were retested in each IR line in which aberrant responses were detected in the primary screen. For these experiments, we grew up the mutants in large-volume flasks to ensure their near-uniform viability, endeavored to prepare single-cell suspensions to better control the size of the infecting inoculum, performed 5–6 independent infections for each and compared responses to those induced by WT Mtb rather than to the average response to all mutants. Resource limitations confined the secondary screen to a subset of 191 hits from the primary screen. These were arbitrarily chosen without regard to quadrant. The following mutants were carried forward into the secondary screen: 55 mutants representing 38% of those in quadrant I of Figure 2B; 10 representing 44% of those in quadrant II; 66 representing 34% of those in quadrant III; and 60 representing 2.6% of those in quadrant IV. Figures 3A and 3B illustrate results for 4 hits identified in the primary screen. In aggregate, 29% of the hits were verified in the secondary screen, distributed as follows. 70% of the mutants tested from quadrant II were verified to dysregulate reporter induction in one or more IR clones. In contrast, the secondary screen confirmed only 25% and 24% of the tested mutants from quadrants I and III, confirmation rates that were comparable to those observed for mutants from quadrant IV. These observations conformed to expectations for false positives from statistical expectations according to the cut-off values chosen and the number of IR clones dysregulated. Among the false positives from the primary screen were 23 mutants that were also false negatives in the primary screen in the sense that they consistently exhibited altered fluorescence induction in the secondary screen that was directionally opposite to that observed in the primary screen. These mutants were carried forward as new positives in the secondary screen.

### Phenotypes of Mtb Mutants in Primary Macrophages and Identification of Mutated Genes

**Figure 4:**
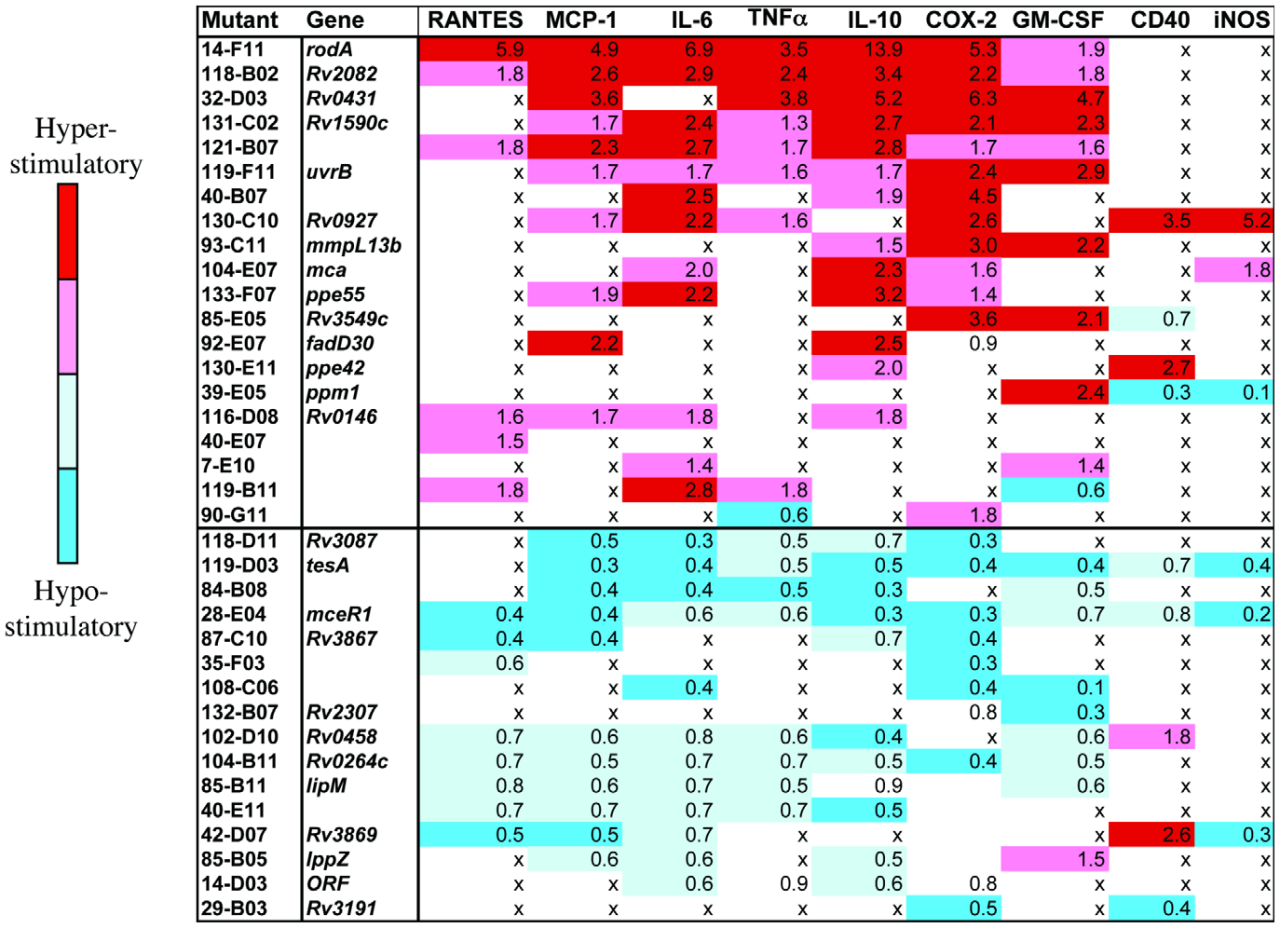
Summary of Host Responses in Mutant-Infected BMD Macrophages. Values represent the relative increase in the immune response as a function of increasing MOI, as compared to the response induced by WT Mtb. Only responses that were statistically different from the WT responses (p<0.05) are quantified. Responses were pooled from three independent experiments, each of which was evaluated over a range of three distinct MOIs. Fold-change >2.0 highlighted in red, 1.4–2.0 in pink, 0.6–0.7 in light blue, and <0.5 in dark blue. “x” means that the response failed to meet statistical significance. Gene names are supplied for strains in which mapping of the insertion site was successful.

The next set of studies put aside IR clones and promoter-reporter constructs in favor of primary bone marrow-derived macrophages and endogenous gene products. We measured transcript levels by qRT-PCR for GM-CSF, GITRL, CD40, COX2, and iNOS and secreted proteins by ELISA for IL-10, RANTES, TNFα, IL-12p40, MCP-1 and IL-6. We tested responses to 3 different MOIs for both WT and mutant strains of Mtb in 3 independent experiments per mutant, used the complete data sets to calculate a single value (“fold change”) for the greater or lesser efficiency of each Mtb mutant in eliciting a given response compared to WT Mtb, and calculated the statistical significance of each fold change, as detailed in the Supplemental Materials and Methods. In these experiments, we studied 53 of the 56 hits verified in the secondary screen. Figure 4 lists the 36 (68%) of these Mtb mutants that elicited statistically significant fold-change values in primary macrophages and were therefore considered verified in the tertiary screen.

We were able to map the site of transposon insertion for 26 of the 36 mutants that confirmed in the tertiary screen (Figure 4). Table 1 lists the genes in which the transposon inserted, without implying that the phenotype necessarily arises from loss of function in the designated gene. To assign function it is necessary to evaluate the possibility of polar effects of each insertion and to complement each disrupted gene with a WT copy; such efforts are beyond the scope of this initial study.

**Table 1:** Identification of Transposon Insertion Site in Mutants with Confirmed Immunoregulatory Phenotypes.

Rv Designation	Gene	Annotation	Phenotype (Hyper- or Hypo-Stimulatory Relative to WT Mtb)
**Lipid Metabolism & Trafficking**			
*Rv1146*	*mmpL13b*	Transmembrane transport protein	Hyper-
*Rv0404*	*fadD30*	Long-chain fatty acyl-AMP ligase	Hyper-
*Rv2928*	*tesA*	Thioesterase	Hypo-
*Rv3087*	*---*	Triacylgleycerol synthase	Hypo-
*Rv2284*	*lipM*	Lipid esterase	Hypo-
*Rv2051*	*ppm1*	Polyprenol-monophosphomannose synthase	Mixed
*Rv0017c*	*rodA*	Cell division protein	Hyper-
**Secreted Proteins/Secretion Systems**			
*Rv3867*	*---*	Conserved hypothetical = > RD1 locus	Hypo-
*Rv3869*	*---*	Conserved hypothetical = > RD1 locus	Mixed
*Rv2608*	*ppe42*	PPE protein	Hyper-
*Rv3006c*	*lppZ*	Lipoprotein	Mixed
*Rv3347c*	*ppe55*	PPE protein	Hyper-
**Transcriptional Regulators**			
*Rv0165c*	*mce1R*	GNTR-family transcriptional repressor	Hypo-
*Rv0431*	*---*	Tuberculin-related peptide	Hyper-
**Intermediary Metabolism**			
*Rv0458*	*---*	Aldehyde Dehydrogenase	Hypo-
*Rv3549c*	*---*	Short-chain type dehydrogenase/reductase	Hyper-
*Rv0927*	*---*	Short-chain type dehydrogenase/reductase	Hyper-
**Miscellaneous**			
*Rv1633*	*uvrB*	DNA repair enzyme	Hyper-
*Rv1082*	*mca*	Mycothiol conjugate amidase	Hyper-
*Rv0146*	---	SAM-dependent methyltransferase	Hyper-
*Rv3191*	---	Transposase	Hypo-
Unannotated	---	ORF between Rv3428 and ppe59	Hypo-
**Conserved Hypothetical**			
*Rv0264c*	---	Conserved Hypothetical	Hypo-
*Rv1590*	---	Conserved Hypothetical	Hyper-
*Rv2307*	---	Conserved Hypothetical	Hypo-
*Rv2082*	---	Conserved Hypothetical	Hyper-

### Phenotype of Mtb Tn:*Rv0431*

**Figure 5:**
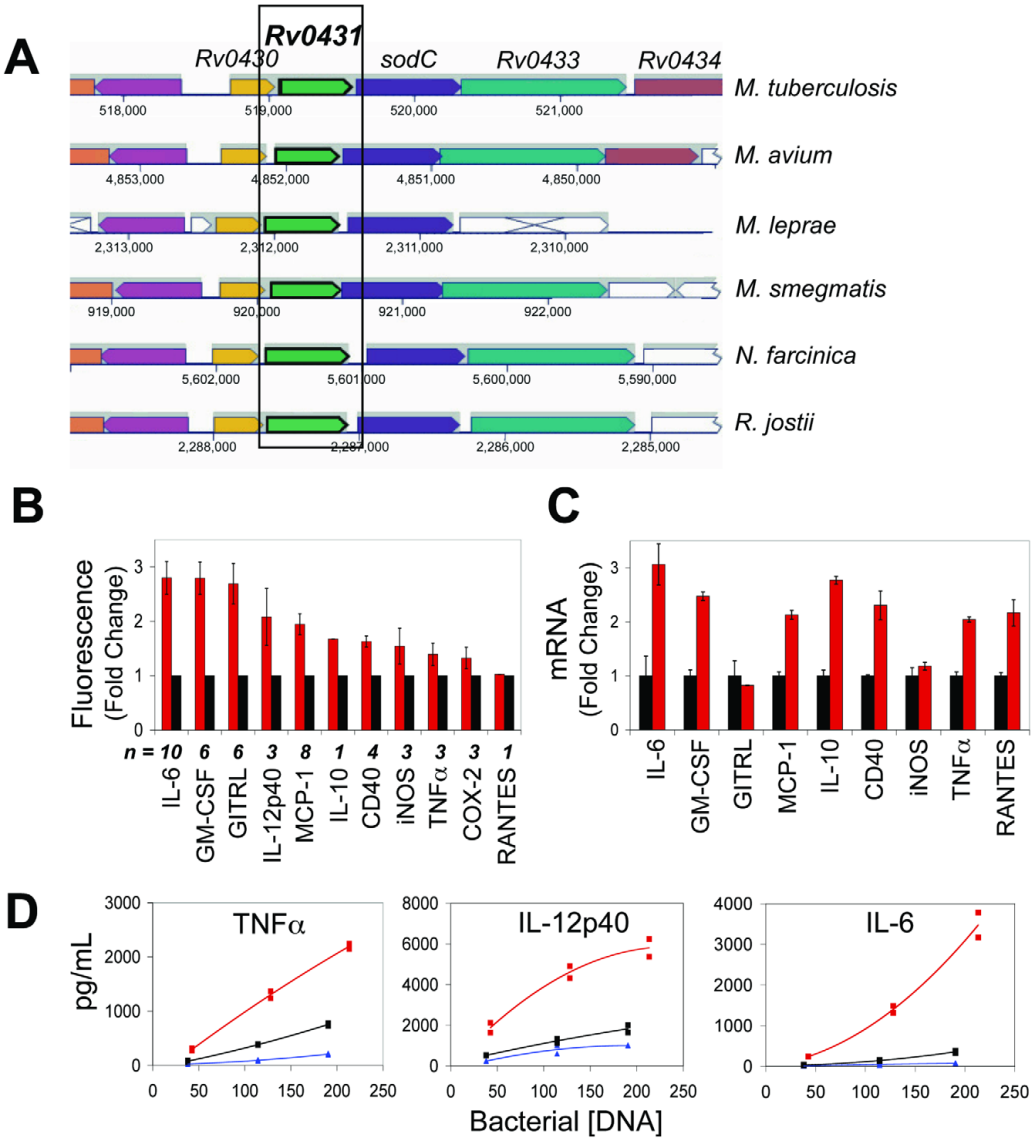
Tn:*Rv0431* Dysregulates Macrophage Immunity. (**A**) Mtb *Rv0431* appears in the context of a suite of genes, including R*v0430*, *sodC*, *Rv0433*, and *Rv0434* which are predicted to be co-transcribed. The members and arrangement of this operon are evolutionarily conserved across other *Mycobacteria* and the related *Actinobacteria* species, *Nocardia* and *Rhodococcus*. (**B**) Average fluorescence induction in IR macrophages by Tn:*Rv0431* (red) or WT Mtb (black) in the number of independent experiments indicated by “n”. Data expressed as the average fold-change relative to induction by WT Mtb ± SE of replicate wells (**C**) WT RAW macrophages were infected withTn:*Rv0431* (red) or WT Mtb (black). Gene induction was assessed at 8 hrs post-infection by qRT-PCR. Data are expressed as mean ± SD of triplicate wells. (**D**) BMD macrophages were infected with WT Mtb (black), Tn:*Rv0431* (red) or Tn:*Rv0431*::*Rv0431* (blue) over a range of input values (bacterial DNA concentration in pg/mL). TNFα, IL-6, and IL-12p40 were measured in the supernatants by ELISA at 26 hours. Responses in duplicate wells were assessed for each input value. Second-order polynomial trend lines were fit to the data using Excel.

We selected one mutant for more extensive characterization of its impact on the host-pathogen interaction. In Mtb Tn:*Rv0431*, the transposon inserted between codon 121 and 122 of *Rv0431*, a gene predicted to encode a 164-amino acid protein of unknown function. Southern analysis of genomic DNA from this mutant confirmed a single transposon insertion site (data not shown). *Rv0431* is the second gene in a putative 5-gene operon that also includes *Rv0430*, *sodC*, *Rv0433*, and *Rv0434*. The putative operon is highly conserved in *Mycobacteria* and other members of the *Actinobacteria* family, including *Nocardia* and *Rhodococcus* species (Figure 5A). The only gene in the putative operon with an assigned function is *sodC*, which encodes a membrane-tethered zinc/copper superoxide dismutase.

In the secondary screen, Tn:*Rv0431* induced higher levels of fluorescence in all IR clones except the RANTES_AmCyan IR line (Figure 5B), the IR clone that gave the fewest verifiable results. We infected WT RAW 264.7 cells with Tn:*Rv0431* or WT Mtb and evaluated induction of nine of the native host genes using qRT-PCR. Infection with Tn:*Rv0431* resulted in greater expression of transcripts for IL-12p40, IL-6, Delta4, CD40, GM-CSF, RANTES, TNFα and MCP-1, but not GITRL or iNOS, than WT Mtb, both with (not shown) or without IFNγ costimulation (Figure 5C). Results were similar at the protein level for the three products tested by ELISA (IL-6, MCP-1 and IL-12p40; data not shown).

Compared to WT Mtb, infection of primary macrophages with Tn:*Rv0431* resulted in secretion of much larger amounts of IL-10, IL-6, MCP-1, IL-12p40 and TNFα as judged by ELISA and higher levels of transcripts for GM-CSF and COX-2 as measured by qRT-PCR (Figure 5D and Figure S3A–B). To confirm that comparable numbers of bacteria were used for the infection, we measured the genomic content of each bacterial strain at each of the three doses administered to macrophages. As shown in Figure 5D, responses increased in a dose dependent manner for each strain but Tn:*Rv0431* consistently induced more vigorous responses when comparable bacterial doses were evaluated. This trend was consistent when responses were regressed against a secondary measure of bacterial input, colony-forming units (determined by plating the input inoculum on enriched solid agar; Figure S3C). Importantly, dysregulation was gene specific, as RANTES, iNOS, and CD40 levels were induced comparably by both WT and Tn:*Rv0431* (Figures S3A–B).

To confirm that disruption of *Rv0431* caused the altered immune responses in macrophages, and to rule out effects due to secondary mutations, expression of *Rv0431* was restored in Tn:*Rv0431* by complementation using the predicted promoter upstream of *Rv0430*. As shown in Figure 5D, genetic restoration of *Rv0431* in Tn:*Rv0431* fully reverted the immunoregulatory phenotype to that of WT Mtb.

**Figure 6:**
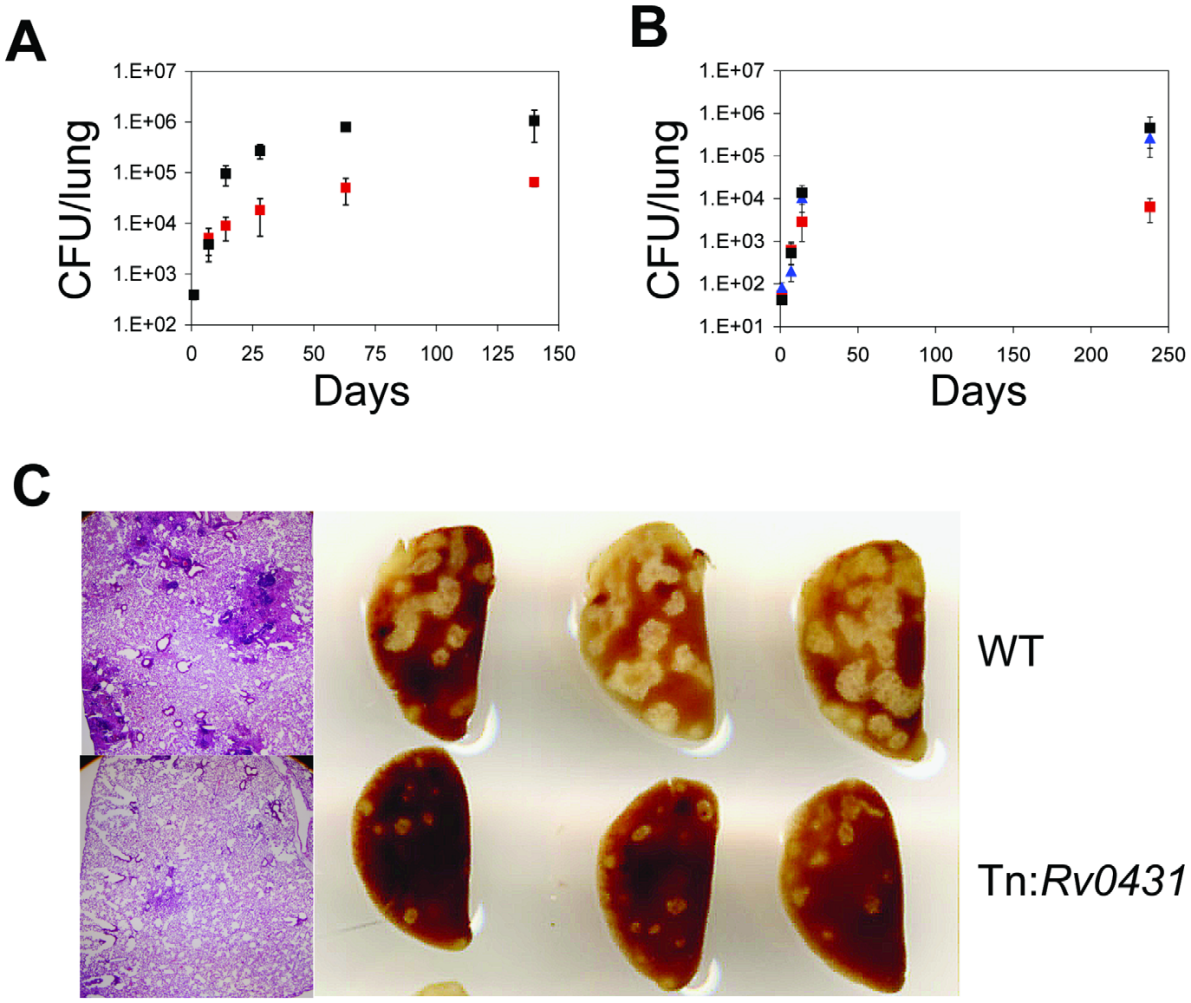
Tn:*Rv0431* is Attenuated In Vivo and Elicits Heightened Immune Responses in the Lung. (**A**) Mice were infected with WT Mtb (black squares) or Tn:*Rv0431* (red squares) via the aerosol route. Bacterial burdens were assessed in the lung at indicated times post-infection. Data points are means ± standard deviations from 3–5 mice. Results are representative of 3 independent experiments. (**B**) As in (A), except Tn:*Rv0431*::*Rv0431* (blue triangles) was included in this experiment. (**C**) Gross pathology (right panel) and hematoxylin and eosin staining of lung sections (40× magnification; left panel) from infected animals. Gross pathology images are from the upper left lobes of 3 mice at day 140 post-infection. Histopathology images are representative sections from the upper left lobes of mice at day 63 post-infection.

Finally, we assessed the phenotype of the Tn:*Rv0431* mutant in vivo by exposing C57BL/6 mice to aerosols of Tn:*Rv0431* or WT Mtb. Both strains replicated at comparable rates during the first week. However, between the first and second week—before the onset of adaptive immunity detectable with conventional assays— Tn:*Rv0431* became markedly attenuated in the lungs of infected animals, with CFU an order of magnitude lower than WT at 2 weeks and 2 orders of magnitude lower than WT by 34 weeks (Figure 6A, B). Genetic complementation of Tn:*Rv0431* with the WT allele restored the bacterial burden to WT levels (Figure 6B). Infection with Tn:*Rv0431* was associated with correspondingly less gross and microscopic pathology than infection with WT Mtb (Figure 6C). A similar level of attenuation was noted in the spleens of Tn:*Rv0431*-infected animals at later time points (Figure S4A). Attenuation in vivo was not due to a general growth defect, as Tn:*Rv0431* grew like WT Mtb in standard 7H9 medium (Figure S5).

Differences in cytokine expression in response to infection by two different strains of Mtb are difficult to interpret when antigenic load is different. However, the nearly identical bacterial loads at one week after infection of mice with Tn:*Rv0431* or WT Mtb allowed an analysis of immune response gene expression in the lungs at this early time point using qRT-PCR, although levels were not yet high enough for detection by ELISA. As shown in Figure S4B, expression of TNFα, IL-6, Lrg-47 and CD40 was significantly higher in lungs of mice infected with Tn:*Rv0431* than WT Mtb.

## Discussion

This study represents one of the most extensive surveys of the Mtb genome to date to identify factors involved in modulating macrophage immunity. Many reports have studied single Mtb genes for their impact on host immunity or multiple Mtb genes for their immunomodulatory effects on single host genes [22], [23], [27]. Here, we screened 10,100 Mtb mutants for their impact on expression of 12 host cytokines, chemokines, enzymes and developmental regulators. We identified 35 Mtb genes with previously unknown roles in host immunity and another 228 Mtb genes that are strong candidates for regulating these host responses. We focused on one gene of unknown function, *Rv0431*, and confirmed its profound role in suppressing TNFα, IL12p40 and IL-6 production in primary macrophages. Mtb lacking Rv0431 grew normally in vitro but poorly in mice and thereafter failed to maintain its numbers during the chronic phase of the infection.

Though important, these findings must be viewed in the context of the substantial technical challenges associated with a screen of this breadth and depth, most notably the sensitivity of the macrophage responses to variations in the bacterial inoculum, which could not be perfectly normalized across 10,100 mutants. Nonetheless, despite a large false positive rate in the primary screen, 68% of the mutants that we subjected to further study were confirmed to dysregulate innate responses of primary macrophages. Confirmatory analyses are required for the other 228 equally promising candidates identified in the primary screen that we have not yet tested in primary macrophages (marked by asterisks in Tables S1, S2, S3, S4).

With the exception of mceR1 [28], the transposon-disrupted genes identified in our screen have not been reported to be involved in immune regulation. However, based on what is known about the interrupted genes, their homologs in other organisms and their neighbors on the Mtb chromosome, we consider possible mechanisms for the observed immunomodulatory properties of these mutants.

Given that lipids are estimated to contribute >40% of the dry weight of the bacillus [29], that some are shed [30] and that others may be among the first molecules on the Mtb surface that macrophages encounter, it is not surprising that many Mtb lipids have well-described roles in modulating host immunity, including phthiocerol dimycoserosates (pDIM), sulfolipids, lipomannans, mycolic acids and phenolic glycolipids [31], [32], [33], [34]. Many of the disrupted genes discovered in our screen have predicted roles in the synthesis, degradation or trafficking of Mtb lipids, including *mmpL13B*, *tesA, fadD30, Rv3087, lipM,* and *ppm1.* For example, *mmpL13B* encodes a putative lipid transporter with homology to other transporters, such as MmpL7 and MmpL8, known to deliver immunomodulatory lipids to the cell wall [35], [36], [37], [38], [39]. Another identified gene, *fadD30,* is predicted to encode a long-chain fatty acyl-AMP ligase involved in lipid synthesis [40]. Deletion of a related enzyme, FadD26, led to a defect in pDIM production associated with enhanced TNFα and IL-6 secretion from macrophages [41]. In addition, the gene immediately downstream of and likely cotranscribed with *fadD30*, *pks6,* is important for polar lipid synthesis [42] and virulence in mice [43], where it plays a role in countering IFNγ-dependent host defenses [44].

Several other genes we identified could play ancillary roles in lipid synthesis or storage. *tesA,* encoding a type II thioesterase, is located immediately upstream of a large virulence cluster involved in pDIM biosynthesis and has a postulated role in the release of mature lipids from the pDIM synthesis machinery [42], [45]. *Rv3087*, codes for a triacylgylcerol synthase within the *mymA* operon, which has been linked to virulence, resistance to host defenses, and the synthesis of mycolic acids, another lipid class with potent and diverse roles in immunological modulation by Mtb [27], [46], [47], [48], [49], [50], [51], [52], [53]. A mutant lacking the *mymA* operon induced significantly less IL-1β, IL-6, RANTES, and MCP-1 in human THP-1 monocyte-like leukemia cells [54], consistent with our findings of diminished immune responses in primary mouse macrophages by the Tn:*Rv3087* mutant. *Ppm1* encodes polyprenol-monophosphomannose synthase, an enzyme involved in the synthesis of the cell wall glycolipids, lipomannan and lipoarabinomannan, both of which function as TLR ligands and as modulators of immune cell function [31].

Several Mtb immunoregulatory genes identified here encode secreted factors (*lppZ*, ppe55, and *ppe42*) or proteins involved in secretion systems (*Rv3867* and *Rv3869*). Rv3869, and perhaps Rv3867, function as part of a multi-protein translocon known as the ESX-1 secretion system, which is involved in the secretion of virulence and immumodulatory factors, including ESAT-6 and CFP-10 [55], [56], [57]. ESX-1-related effects on host immunity include modulation of inflammation, apoptosis, necrosis, phagosomal maturation, phagosomal escape, membrane/cell lysis, tissue invasion, cell-to-cell spread and virulence. The secreted proteins LppZ, PPE55, and PPE42 are dominant B-cell antigens in tuberculosis patients [58], [59], [60]. Other PE/PPE family proteins are known to modulate cytokine production and apoptosis in T-cells and macrophages [61], [62], [63], [64], [65].

To illustrate the potential of this approach to provide insights into the immunobiology of experimental tuberculosis, we focused on Rv0431 because nothing has been reported about this gene, yet its disruption had a striking effect on macrophage production of cytokines in vitro and on tuberculosis in the mouse. Disruption of Rv0431 led to enhanced fluorescence induction in 11 of the 12 IR macrophage cell clones and robust hyperinflammatory responses in mutant-infected primary macrophages. In vivo, Tn:*Rv0431* became markedly attenuated between day 7 and 14 post-infection and, at later time points, the reduction in bacterial load was mirrored by a decrease in accumulation of host lymphoid and myeloid cells in the lungs. Given that Tn:*Rv0431* has no growth defect in standard broth in vitro, these findings demonstrate a role for Rv0431 in Mtb virulence. Moreover, attenuation in vivo was preceded by suggestive evidence for an increased inflammatory responses in the lung (TNFα, IL-6, TGFβ, LRG-47 and CD40) commonly associated with macrophage or DC responses. The attenuation of Tn:*Rv0431* in vivo is noteworthy in that neither SodC nor Rv0433 appear to be required for full virulence in mice [66]. Nevertheless, SodC is markedly upregulated upon infection of human macrophages and is important for the survival of Mtb in activated murine macrophages, where it likely functions in the defense against reactive oxygen species [67], [68]. Collectively, these findings implicate Rv0431 and its neighboring genes as important Mtb virulence factors and highlight the need for additional studies to explore the function(s) of this gene cluster.

To our knowledge, this is the first description of a biological role for Rv0431. Though its cellular function remains unknown, two short nucleotide stretches in the C-terminus of Rv0431 have low homology to sequences in other bacterial proteins annotated as cell envelope-related or LytR-family transcriptional regulators. In other organisms, LytR-related transcriptional regulators have important roles in peptidoglycan formation, cell wall maintenance, biofilm formation, β-lactam resistance and stress tolerance [69], [70]. Further study will clarify whether Rv0431 is a transcriptional regulator.

BCG is the only vaccine now available against tuberculosis but shows poor efficacy in preventing pulmonary tuberculosis in adults [71], [72]. The limited efficacy of BCG is not surprising, given that infection with Mtb itself does not routinely lead to protective immunity against re-infection [3], [4], [5]. It is a tall order for a vaccine to generate better immunity than native infection. Most experimental tuberculosis vaccines use one or two antigens from Mtb [73]. The work described here is a first step toward testing a complementary strategy—to subtract a small number of antigens from Mtb, so that the immune response it elicits is more protective. After we or others study additional mutants identified in the present study, it is our hope that mice can be vaccinated with a single strain engineered to include several such mutations, along with several that impose auxotrophies to prevent the mutant from replicating in the host [74].

## Materials and Methods

### Ethics Statement

Studies with mice followed a protocol approved by the Weill Cornell Medical College Institutional Animal Care and Use Committee (#0705-617) and were carried out in strict accordance with the recommendations in the Guide for the Care and Use of Laboratory Animals of the National Institutes of Health.

### Mice

Eight-week old female C57BL/6 mice from Jackson Laboratories (Bar Harbor, Maine) were housed in specific-pathogen-free facilities at Weill Cornell Medical College and Rockefeller University.

### Mycobacterial Cultures

H37Rv Mtb was from ATCC. The *mce*1 mutant [26] and its parent strain (Erdman) were kindly provided by S. Ehrt, Weill Cornell Medical College. The mutant library and Mtb growth in vitro were as described [25]. Individual mutants were frozen at -80 C in 15% glycerol/7H9 in 96-well plates; library mutants were thawed and resuspended immediately prior to infection of IR macrophages, as described below.

### Infection of IR Macrophage Clones

IR macrophages were cultured at 37°C with 5% CO_2_ in Dulbecco's Modified Eagle's Medium supplemented with 10% heat-inactivated FBS, 2 mM L-glutamine, 1 mM sodium pyruvate, and 1 M HEPES (cDMEM). For fluorescence assays, 10^4^ total cells were seeded in the inner 60 wells of 96-well microscopy plates and cultured overnight. For the primary screen, three IR macrophage clones (each expressing either YFP, DsRed, or AmCyan) were seeded together in each well and simultaneously infected with each mutant in the Mtb library. IR macrophages were treated with 2.5 ng/mL IFNγ (Genentech) and infected with 1 µL of each Mtb mutant. Because of differences in bacterial viability after the freeze-thaw cycle required to store the library, the actual MOI (as determined by retrospective plating for CFUs on 7H9 agar) varied considerably across the strains. At 22–24 hrs post-infection, cells were fixed with 4.2% paraformaldehyde in PBS for 2 hrs, washed twice with PBS and stained with 2 µg/mL Hoechst 33342 in PBS. For each round of infection, control wells on separate plates were studied in medium alone to confirm low basal fluorescence and after exposure to 2.5 ng/mL IFNγ to confirm responsiveness to a stimulus.

### Confirmation of Hits in IR Macrophage Clones

Hits from the primary screen were re-cloned on 7H11/OADC agar, then grown to early-log phase in 7H9/ADN. Clumps were removed by centrifugation in PBS-0.05% Tween80 to generate single-cell suspensions. Bacterial concentration was back-calculated from OD_580_ measurements and bacteria were resuspended in cDMEM for infection of macrophages. Individual (rather than pooled) IR macrophage clones were infected with each mutant or WT Mtb at MOI  = 10 and fluorescence induction was measured as described above. Mutants that induced ≥1.4-fold more or ≤0.7-fold less fluorescence than WT Mtb were considered “confirmed” with respect to the IR clones and were then tested in primary macrophages.

### Hit Validation in Primary Bone Marrow-Derived (BMD) Macrophages

BMD were derived as described [12], except antibiotics were omitted. 10^5^ macrophages were infected with single-cell suspensions of pDIM+ WT Mtb or mutant strains at target MOIs of 1, 3 and 5. Tn:*Rv0431* and a pDIM-deficient strain were included as controls for higher and lower induction, respectively. Uninfected cells served as a negative control. Macrophage responses were assessed by qRT-PCR at 8 hrs or by ELISA (RnD Systems) at 24 hrs post-infection. Two intra-assay replicates per MOI were included for every condition and 2–3 independent experiments were performed for each mutant. Bacterial input was determined retrospectively by plating serial dilutions on 7H11/OADC agar and enumerating CFUs.

### mRNA Isolation and qRT-PCR

Macrophages were lysed in TRIzol reagent (Invitrogen). Bromochloropropane was added, then RNA was isolated using the MagMAX-96 Total RNA Isolation Kit including treatment with MagMAX TURBO DNase (Ambion). ∼300 ng RNA was reverse transcribed using 50 units MuLV reverse transcriptase, 2.5 µM oligo-d(T)_16_ primer and 20 units RNase Inhibitor (Applied Biosystems). mRNA expression levels were evaluated by qRT-PCR using gene-specific primers and Taqman probes labeled with FAM and BHQ on the 5′- and 3′-ends, respectively. Amplifications were performed on the ABI Prism 7900HT sequence detection system (PerkinElmer). Threshold values were set in SDS 1.0 software (Applied Biosystems) such that CT values were derived from the early linear phase. The abundance of gene-specific mRNAs was quantified relative to expression of the housekeeping gene GAPDH, according to the formula: Relative mRNA Induction  = 2^CT(GAPDH)–CT(GENE)^.

### Identification of Transposon Insertion Sites

Genomic DNA was purified from select mutants and the transposon insertion site was identified by sequencing.

### Mouse Infections

Mice were infected by aerosolizing Mtb suspensions in PBS-0.05% Tween80 in a Middlebrook airborne infection apparatus (Glas-col, Terre Haute, Ind.). Mice were euthanatized with CO_2_ and the right and lower left pulmonary lobes were homogenized in PBS. Serial dilutions were plated on 7H11/OADC agar for CFU determination. The upper left lobe was divided for mRNA extraction and histology (Supplemental Methods).

## Supporting Information

Figure S1 **Evaluation of IR candidates by fluorescence microscopy.** For each IR line, ∼30 clonal populations were evaluated by fluorescence microscopy for fluorescence induction under resting or stimulatory conditions. Clones that showed (a) minimal fluorescence under basal conditions, (b) maximal induction upon stimulation with IFNγ (2.5 ng/mL) or LPS (18 ng/mL), and (c) minimal cell-to-cell heterogeneity among fluorescent cells, were chosen for use in the primary screen. Three such clones, from the YFP_IL-10 IR line, are shown here as an example.
(TIF)

Figure S2 **Fluorescence induction is decreased following infection with a hypostimulatory Mtb mutant.** (A) Fluorescence induction in the MCP-1_DsRed and IL-6_YFP IR clones in response to infection with WT (black bars) or mce1-deficient (red bars) Mtb. Unstimulated cells were included as a control (grey bar). (B) Secretion of native MCP-1 and IL-6 by WT RAW264.7 cells in response to infection with WT or mce1-deficient Mtb, as assessed by ELISA. Data are representative of two independent experiments. Bars represent mean and standard deviations of triplicate wells. In experiments measuring IL-6 secretion and IL-6_YFP fluorescence, macrophages were costimulated with 2.5 ng/mL IFNγ.
(TIF)

Figure S3 **BMD macrophages were infected with WT Mtb (black) or Tn:** ***Rv0431*** **(red) over a range of bacterial inputs.** Responses in duplicate wells were assessed for each strain at each input value. Second-order polynomial trend lines were fit to the data using Excel. (A) and (B) are from a single representative experiment in which responses were regressed against bacterial DNA concentration (ng/uL). (**A**) Cytokines in the supernatants were measured by ELISA at 26 hours. (**B**) Host mRNA expression was measured by qRT-PCR at 8 hours. (**C**) As in (A), except that responses were regressed against the MOI, as determined by plating for CFUs on enriched solid agar.
(TIF)

Figure S4 **Tn:** ***Rv0431*** **is Attenuated** ***In Vivo*** **and Elicits Heightened Immune Responses in the Lung.** Mice were infected with WT Mtb (black) or Tn:*Rv0431* (red) via the aerosol route. (**A**) Bacterial burdens in the spleen at days 14, 28, 63, and 140 post-infection. Data points are means and standard deviations from 3-5 mice. Results are representative of two independent experiments. (**B**) Expression of host genes in the lungs of infected animals at day 7, as determined by qRT-PCR. Data are expressed as fold-change relative to responses induced by WT Mtb. Bars represent the mean and SD from 5 mice. Asterisk denotes p < 0.05. Results are representative of two independent experiments.
(TIF)

Figure S5 **Tn:** ***Rv0431*** **Does Not Have a Growth Defect In Enriched Liquid Culture.** WT Mtb (black) and Tn:*Rv0431* (red) were grown in 7H9-ADN liquid media for 14 days at 37 C. The optical density was measured at indicated intervals at 580 nm as a read-out for culture density.
(TIF)

Table S1 **Mutants from the Primary Screen that Dysregulated One IR Clone with |SR| > 3.5 (Quadrant I in** **Figure 2B** **).** Values represent the SR of the response induced by each mutant (rows) in each of the twelve IR clones (columns) in the primary screen. SR > 3.5 in red; SR < -3.5 in blue. Mutants marked by an asterisk were not evaluated in follow-up studies.
(XLS)

Table S2 **Mutants from the Primary Screen that Dysregulated Two IR Clones with an Average |SR| > 3.5 (Quadrant I in** **Fig 2B** **).** Values represent the SR of the response induced by each mutant (rows) in each of the twelve IR clones (columns) in the primary screen. SR > 3.5 in red; 2.0 < SR < 3.5 in pink; -2.0 > SR > -3.5 in light blue; and SR < -3.5 in dark blue. The average |SR| (for all responses with |SR| > 2) is quantified for each mutant in the second column from the right. In the far right column, “+” denotes a mutant that induced hyperstimulatory responses, “–” denotes hypostimulatory, and “M” denotes mixed response across IR clones. Mutants marked by an asterisk were not evaluated in follow-up studies.
(XLS)

Table S3 **Mutants from the Primary Screen that Dysregulated >2 IR Clones with an Average |SR| > 3.5 (Quadrant II in** **Figure 2B** **).** Values represent the SR of the responses induced by each mutant in each of the twelve IR clones in the primary screen. SR > 3.5 in red; 2.0 < SR < 3.5 in pink; -2.0 > SR > -3.5 in light blue; and SR < -3.5 in dark blue. The average |SR| (for all responses with |SR| > 2) is quantified for each mutant. Second column from the right indicates the number of IR clones with an |SR| > 2.0. In the last column, “+” denotes hyperstimulatory responses, “–” denotes hypostimulatory responses and “M” denotes mixed responses across IR clones. Mutants marked by an asterisk were not evaluated in follow-up studies.
(XLS)

Table S4 **Mutants from the Primary Screen that Dysregulated >2 IR Clones with an Average 2.0 < |SR| < 3.5 (Quadrant III in** **Fig 2B** **).** Values represent the SR of the responses induced by each mutant in each of the twelve IR clones in the primary screen. SR > 3.5 in red; 2.0 < SR < 3.5 in pink; -2.0 > SR > -3.5 in light blue; and SR < -3.5 in dark blue. The average |SR| (for all responses with |SR| > 2) is quantified for each mutant. Second column from the right indicates the number of IR clones with an |SR| > 2.0. In the last column, “+” denotes hyperstimulatory responses, “–” denotes hypostimulatory responses and “M” denotes mixed responses across IR clones. Mutants marked by an asterisk were not evaluated in follow-up studies.
(XLS)
