# Supplementary figures and images for: Genome-Wide Screen for Mycobacterium tuberculosis Genes That Regulate Host Immunity
Source: PLoS One. 2010 Dec 10;5(12):e15120. doi: 10.1371/journal.pone.0015120 (PMC3000826; doi:10.1371/journal.pone.0015120)

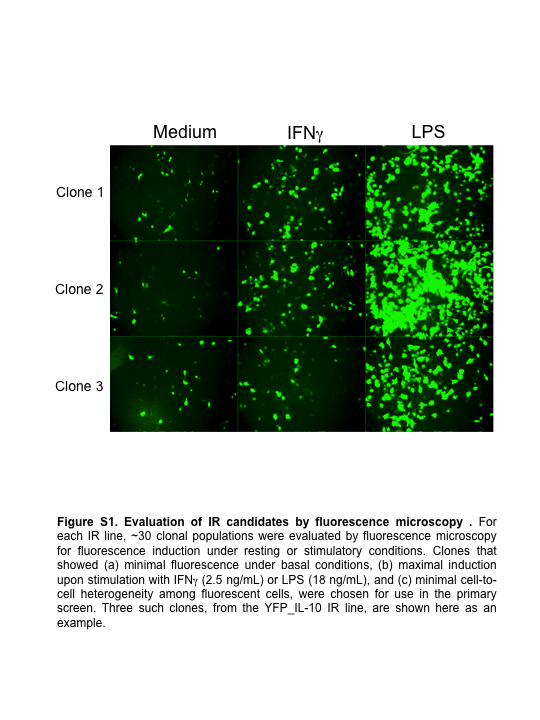

Supplement: Figure S1 — Evaluation of IR candidates by fluorescence microscopy. For each IR line, ∼30 clonal populations were evaluated by fluorescence microscopy for fluorescence induction under resting or stimulatory conditions. Clones that showed (a) minimal fluorescence under basal conditions, (b) maximal induction upon stimulation with IFNγ (2.5 ng/mL) or LPS (18 ng/mL), and (c) minimal cell-to-cell heterogeneity among fluorescent cells, were chosen for use in the primary screen. Three such clones, from the YFP_IL-10 IR line, are shown here as an example. (TIF) [file pone.0015120.s001.tif]

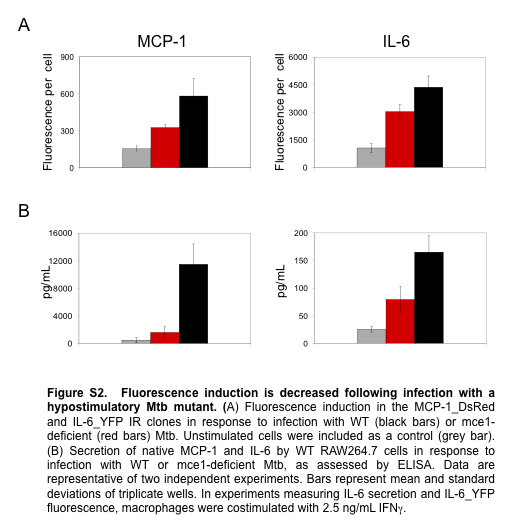

Supplement: Figure S2 — Fluorescence induction is decreased following infection with a hypostimulatory Mtb mutant. (A) Fluorescence induction in the MCP-1_DsRed and IL-6_YFP IR clones in response to infection with WT (black bars) or mce1-deficient (red bars) Mtb. Unstimulated cells were included as a control (grey bar). (B) Secretion of native MCP-1 and IL-6 by WT RAW264.7 cells in response to infection with WT or mce1-deficient Mtb, as assessed by ELISA. Data are representative of two independent experiments. Bars represent mean and standard deviations of triplicate wells. In experiments measuring IL-6 secretion and IL-6_YFP fluorescence, macrophages were costimulated with 2.5 ng/mL IFNγ. (TIF) [file pone.0015120.s002.tif]

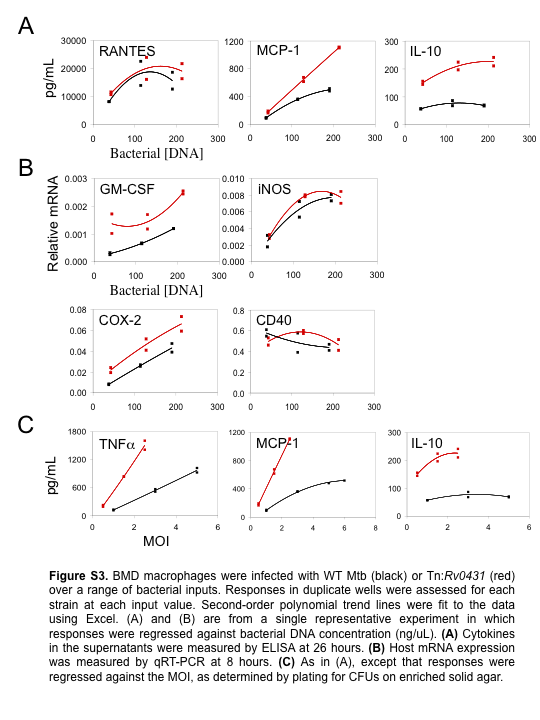

Supplement: Figure S3 — BMD macrophages were infected with WT Mtb (black) or Tn: Rv0431 (red) over a range of bacterial inputs. Responses in duplicate wells were assessed for each strain at each input value. Second-order polynomial trend lines were fit to the data using Excel. (A) and (B) are from a single representative experiment in which responses were regressed against bacterial DNA concentration (ng/uL). (A) Cytokines in the supernatants were measured by ELISA at 26 hours. (B) Host mRNA expression was measured by qRT-PCR at 8 hours. (C) As in (A), except that responses were regressed against the MOI, as determined by plating for CFUs on enriched solid agar. (TIF) [file pone.0015120.s003.tif]

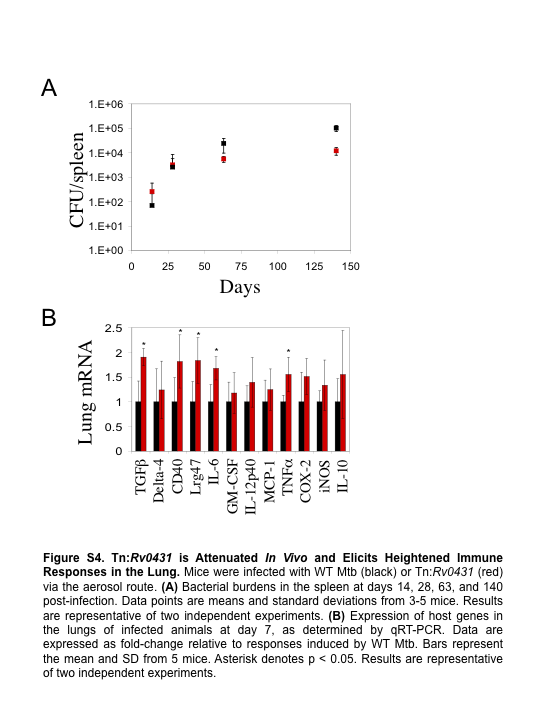

Supplement: Figure S4 — Tn: Rv0431 is Attenuated In Vivo and Elicits Heightened Immune Responses in the Lung. Mice were infected with WT Mtb (black) or Tn:Rv0431 (red) via the aerosol route. (A) Bacterial burdens in the spleen at days 14, 28, 63, and 140 post-infection. Data points are means and standard deviations from 3-5 mice. Results are representative of two independent experiments. (B) Expression of host genes in the lungs of infected animals at day 7, as determined by qRT-PCR. Data are expressed as fold-change relative to responses induced by WT Mtb. Bars represent the mean and SD from 5 mice. Asterisk denotes p < 0.05. Results are representative of two independent experiments. (TIF) [file pone.0015120.s004.tif]

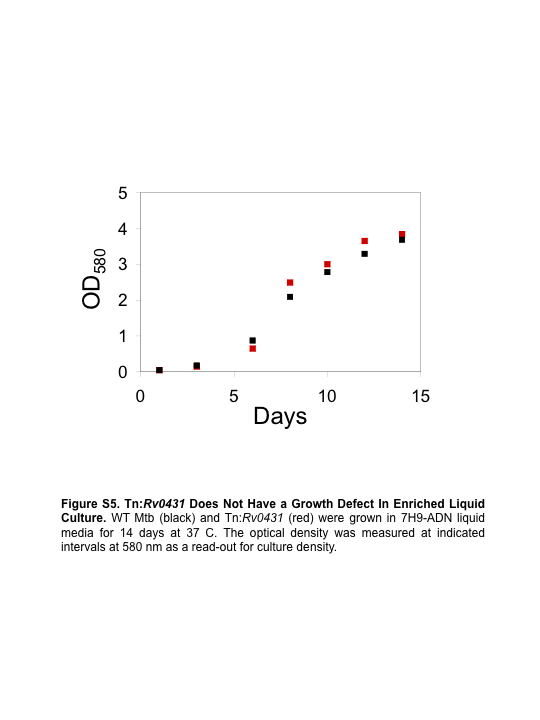

Supplement: Figure S5 — Tn: Rv0431 Does Not Have a Growth Defect In Enriched Liquid Culture. WT Mtb (black) and Tn:Rv0431 (red) were grown in 7H9-ADN liquid media for 14 days at 37 C. The optical density was measured at indicated intervals at 580 nm as a read-out for culture density. (TIF) [file pone.0015120.s005.tif]
